# Development of a metric Healthy Eating Index-2015 and comparison with the Healthy Eating Index-2015 for the evaluation of dietary quality

**DOI:** 10.3389/fnut.2022.952223

**Published:** 2022-08-23

**Authors:** Jan Kohl, Vivien Hohberg, Pascal Hauff, Céline Lang, Oliver Faude, Albert Gollhofer, Daniel König

**Affiliations:** ^1^Department of Sport and Sport Science, University of Freiburg, Freiburg im Breisgau, Germany; ^2^Department of Sport, Exercise and Health, University of Basel, Basel, Switzerland; ^3^Department of Sport Science, Institute for Nutrition, Exercise and Health, University of Vienna, Vienna, Austria; ^4^Department of Nutritional Sciences, Institute for Nutrition, Exercise and Health, University of Vienna, Vienna, Austria

**Keywords:** Healthy Eating Index (HEI), food database, dietary data, dietary quality, HEI-2015, metric data analysis, diet and chronic disease

## Abstract

**Background:**

Diet quality indices can provide important information about relationships between diet and health independent of energy balance. The Healthy Eating Index-2015 (HEI-2015) is widely used and has been extensively evaluated. However, due to imperial units the HEI-2015 is difficult to apply in countries with metric systems. Our objective was to develop a metric version of the HEI-2015 and compare it to the original. The metric Healthy Eating Index-2015 (mHEI-2015) is intended to simplify the application of a dietary quality index in countries using the metric system.

**Methods:**

We developed a metric database logic following the methodology of the HEI-2015, which allows the application to metric databases and was applied to Food Patterns Equivalents Database (FPED). The HEI-2015 was calculated for the National Health and Nutrition Examination Survey (NHANES) 2017-2018 and the scoring standards for each component of the mHEI-2015 was calibrated against it. For the assessment of agreement between indices, HEI-2015 and mHEI-2015 were calculated for NHANES 2015-2016 and a Bland–Altman plot was created.

**Results:**

Healthy Eating Index-2015 and mHEI-2015 for the NHANES 2015-2016 averaged 52.5 ± 13.5 and 52.6 ± 13.2, respectively. The total scores as well as component scores of the indices were strongly correlated. The Bland–Altman plot revealed a high agreement of the total scores. An illustrated analysis of six different menu plans showed only minor differences between the HEI-2015 and mHEI-2015 component scores.

**Conclusion:**

The mHEI-2015 allows for superior analysis of metric dietary data to better examine the relationship between chronic diseases and diet. The streamlined metric methodology enables straightforward application to metric food databases and thus the development of country-specific indices.

## Introduction

It is widely accepted that a healthy and high-quality diet contains a high percentage of health beneficial components such as fiber, unsaturated fatty acids or polyphenols and a low percentage of potentially harmful or unfavorable components like added sugars, saturated fatty acids or sodium (1–5). Additionally, all essential nutrients must be supplied in sufficient quantities, which is ensured by a varied and balanced diet. This balance is achieved mainly through the right amount of certain food groups (6). Prospective studies have found associations between intakes of various food groups and mortality as well as the risk of chronic disease (7–10). For example, a high consumption of fruits, vegetables, or dairy products is associated with lower mortality, while red and processed meats increase mortality (7). In this context, many food groups show a non-linear relationship (7), suggesting the importance of a balanced diet. Nutritional concepts such as the Mediterranean diet or the Dietary Approaches to Stop Hypertension (DASH) diet rely on this balance by providing specific intake recommendations for individual food groups (11, 12) and have proven to be effective in prevention and therapy of non-communicable diseases such as type 2 diabetes or cardiovascular disease (13–16).

Due to its multidimensionality, the evaluation of health-related aspects of diets cannot only be based on macronutrients or individual markers (17). Recently, more and more attention has been paid to the quality of macronutrients (1, 2, 18, 19). To evaluate macronutrient and dietary quality, analyses other than macronutrient distribution are needed. One approach is to use dietary quality scores to consider multiple aspects instead of single markers. There are numerous *a priori* indices for assessing dietary quality (20). These are characterized by being based on the current nutrition knowledge, while in the *a posteriori* approach dietary data is categorized based on statistical analyses (21). Thus, *a priori* indices as compared to *a posteriori* indices are reproducible in different populations because of their causally proven relationships of dietary components (20, 22, 23). One of the most widely used *a priori* index to measure dietary quality is the American version of the Healthy Eating Index (HEI), which was first established by the United States Department of Agriculture (USDA) in 1995 (24) and has received three updates (25–27). These versions of the HEI allow to assess the quality of diet in its entirety and have been adapted to current insights on nutrition. In the American versions of the HEI (original version, HEI-2005, HEI-2010), high quality has shown to be associated with lower all-cause mortality, cardiovascular incidence and mortality, cancer incidence and mortality, type 2 diabetes, and lower mortality among cancer survivors (28, 29). The most recent version of the HEI (HEI-2015) was published in 2018 (27). Like the previous versions of the HEI, the HEI-2015 is composed of food groups and nutrient indicators that score for adequacy or moderation. The 13 categories of the HEI-2015 are related to the energy consumption, ensuring that a high or low energy consumption does not lead to a bias of the resulting scores (20, 27). This allows the assessment of dietary quality even at lower energy intakes, such as in children, in contrast to other indices with fixed recommendations (e.g., HEI-1995 or AHEI-2010). The dietary quality thus retains its comparability even between age groups as shown in the Dietary Guidelines for Americans 2020-2025 (30). In recent years, the HEI-2015 was shown to be associated with lower all-cause mortality and risk of chronic disease such as cardiovascular disease, type 2 diabetes, and several types of cancer (31–34).

The worldwide use and continuous development of the HEI makes this *a priori* index an essential tool to study the relationship between diet and chronic diseases (28, 29, 34, 35). In addition to the use of the HEI in observational studies, the use in intervention studies for cardiometabolic risk conditions is an emerging approach (36). The imperial system of units of the HEI complicates its utilization in countries with metric system. Most countries in the world follow the metric system, thereby limiting the international comparability of dietary data and the use of the HEI. Even the supposedly simple translation of servings, cups and grams presents numerous difficulties and limits comparability (17). Thus, the application of the HEI-2015 or its predecessors is currently linked to the Food Patterns Equivalent Database (FPED) with stored equivalents. However, using the American FPED database in place of a corresponding national food database neglects national consumption patterns and may result in bias in the analysis. For countries with metric system, the use of FPED in combination with HEI is consequently to be considered critical. Therefore, new methods are needed to guarantee high comparability with the highest possible data accuracy.

The objective of this study was to develop a metric version of the HEI-2015 (mHEI-2015) and compare it to the original HEI-2015. The metric adaption and simplified methodology enables the application of the mHEI-2015 with food databases worldwide. Furthermore, the metric version of the HEI-2015 simplifies the analysis of collected metric dietary data and weighed food records. In the future, country-specific adaptations of the HEI-2015 could be created based on the methodology developed in this paper and linked to the corresponding national food database.

## Materials and methods

**FIGURE 1:**
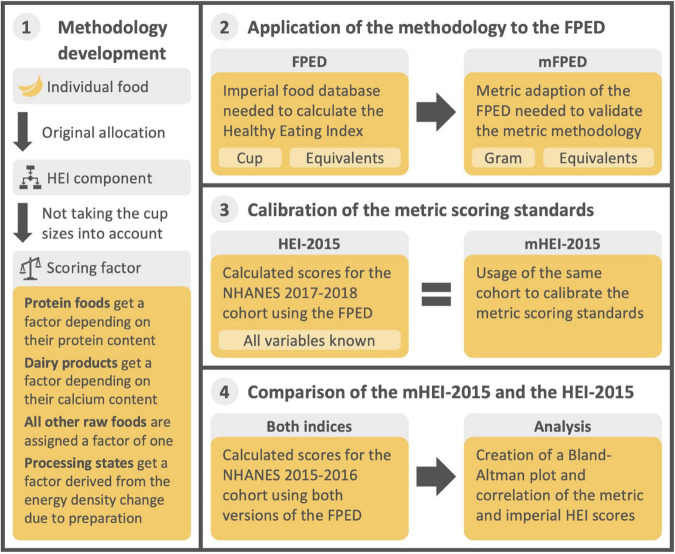
Graphical summary of the methodological approach and the corresponding data basis for the development and comparison of the mHEI-2015. HEI, Healthy Eating Index, FPED, Food Patterns Equivalent Database, mFPED, metric Food Patterns Equivalent Database, mHEI-2015, metric Healthy Eating Index-2015, NHANES, National Health and Nutrition Examination Survey.

In the following, the methodological procedure for the development and comparison of the mHEI-2015 is explained step by step. Figure 1 summarizes the procedure and the data basis.

### The development of the database methodology

The HEI-2015 refers to cup and ounce equivalents. Hence, dietary data can only be evaluated using the corresponding food database. To develop and apply a metric database methodology that is independent of a specific food database, three related food databases were used. The Food and Nutrient Database for Dietary Studies (FNDDS) 2017-2018 contains 7,083 foods and beverages and their nutrient composition. The Food Patterns Equivalent Database (FPED) 2017-2018 is an extension to the FNDDS and provides cup and ounce equivalents for each HEI-2015 component. The majority of the FNDDS foods consist of multiple ingredients. The total of 2,322 ingredients can be found in the Food Patterns Equivalents Ingredient Database (FPID) 2017-2018 and is linked to the FPED 2017-2018 through a recipe database. Because the methodology underlying imperial databases depends on cup sizes, it is impossible to use it to expand metric databases without corresponding cup sizes (37). Therefore, a database methodology that does not rely on cup size, but follows the FPED 2017-2018 methodology was developed for this study. Consequently, the equivalent principle, that compensates for energy density change due to preparation (for fruits, vegetables, and grains) and differences in nutrient content (for total protein foods, seafood and plant proteins, and dairy) was adopted. For components that exclusively represent nutrients (fatty acids, sodium and added sugars) no changes were made as they are already measured in metric units. Where possible, the methodology has been simplified while maintaining maximum comparability with the methodology described in the FPED Methodology and User Guide 2017-2018 (37).

### Application of the methodology to the Food Patterns Equivalents Database

Due to the simplification and translation of the methodology, differences in scoring have emerged. Therefore, the imperial consumption recommendations (standard for maximum score) in each component are no longer compatible with the developed methodology. The scoring standards of the HEI-2015 can be found elsewhere (27). Consequently, a metric and imperial version of the same food database is necessary to set metric values for the maximum score of a component of the mHEI-2015 by means of a calibration, as well as to compare the indices. To accomplish this, the FPED 2017-2018 and the FPED 2015-2016 were revised with the metric methodology from the first step. Therefore, the methodology was applied to all ingredients from the associated FPIDs (37, 38). Since not all foods in the FPIDs are single food items, appropriate recipes had to be reconstructed. This was achieved by assigning single food items according to the cup equivalents. As the information on both the ingredients and the menus was available in cup equivalents, it was possible to determine the portions without estimating. With the revised FPIDs the public recipe database was used to create the metric FPEDs.

### Calibration of Healthy Eating Index-2015 scoring standards to metric scoring standards

To set the metric standards for the maximum score (consumption recommendations) of each modified component of the mHEI-2015, a calibration was performed. For the calibration, dietary data of the National Health and Nutrition Examination Survey (NHANES) 2017-2018 were used (39), as the data are representative and compatible with FPED. NHANES is a national survey that examines nutrition on a 2-year cycle and the data are freely available for scientific use. The data acquisition in NHANES 2017-2018 and NHANES 2015-2016 cohort used in the comparison was performed by 24-h dietary recall using the validated Automated Multiple Pass Method (40). For the calibration, 1- and 2-day dietary records were used (*n* = 7641). Dietary data from children younger than 2 years were excluded (*n* = 511) as in the HEI 2015 evaluation (34). For the remaining 7,130 subjects, the consumption in each component was calculated for the HEI-2015 and mHEI-2015 using the corresponding FPED. From the consumption of a component of the HEI-2015, the proportion at which the maximum score standard was met on average could be calculated. For calibration, it was assumed that the metric consumption in the component corresponded to the same proportion of the standard for the maximum score. For the refined grains component, the metric standard for minimum score was calculated using the calibrated metric standard for maximum score and the ratio of the HEI-2015 standard for minimum and maximum scores. The metric standard for maximum score were determined by the following formulas:

**Formula for dairy and protein components (all values per 1000 kcal):**


m⁢e⁢t⁢r⁢i⁢c⁢s⁢c⁢o⁢r⁢i⁢n⁢g⁢s⁢t⁢a⁢n⁢d⁢a⁢r⁢d=c⁢o⁢n⁢s⁢u⁢m⁢e⁢d⁢p⁢r⁢o⁢t⁢e⁢i⁢n⁢[g]⁢o⁢r

$$c\,a\,l\,c\,i\,u\,m\,\left[m\,g\right]\times \frac{i\,m\,p\,e\,r\,i\,a\,l\,s\,c\,o\,r\,i\,n\,g\,s\,t\,a\,n\,d\,a\,r\,d}{c\,o\,n\,s\,u\,m\,e\,d\,c\,u\,p\,o\,r\,o\,u\,n\,c\,e\,e\,q\,u\,i\,v\,a\,l\,e\,n\,t\,s}$$

**Formula for the remaining food group-based components (all values per 1000 kcal):**


m⁢e⁢t⁢r⁢i⁢c⁢s⁢c⁢o⁢r⁢i⁢n⁢g⁢s⁢t⁢a⁢n⁢d⁢a⁢r⁢d=c⁢o⁢n⁢s⁢u⁢m⁢e⁢d⁢g⁢r⁢a⁢m

$$e\,q\,u\,i\,v\,a\,l\,e\,n\,t\,s\times \frac{i\,m\,p\,e\,r\,i\,a\,l\,s\,c\,o\,r\,i\,n\,g\,s\,t\,a\,n\,d\,a\,r\,d}{c\,o\,n\,s\,u\,m\,e\,d\,c\,u\,p\,o\,r\,o\,u\,n\,c\,e\,e\,q\,u\,i\,v\,a\,l\,e\,n\,t\,s}$$

### Comparison of the Healthy Eating Index-2015 and metric Healthy Eating Index-2015

To examine comparability at the collective and individual levels as well, we calculated the mHEI-2015 and HEI-2015 of the NHANES 2015-2016 cohort (39). For 8,505 subjects in the cohort, at least 1 day of dietary records were available. Dietary data from children younger than 2 were excluded (*n* = 583) as in the HEI-2015 evaluation (34) and in the previous calibration. A total of 7922 subjects with 1- or 2-days dietary record were included in the analysis. To review the agreement of the HEI-2015 and mHEI-2015, a Bland-Altman plot was created including the mean bias together with the 95% limits of agreement (calculated as ± 1.96 × the standard deviation of the difference). Furthermore, the correlation between the total scores for the HEI-2015 and mHEI-2015 was calculated using Pearson’s r. For the components of both indices, the correlation was determined using Spearman’s rho due to the lack of normal distribution. Calculation of HEI-2015 and mHEI-2015 scores and calibration of mHEI-2015 were performed using Microsoft Excel (Version 2108). The statistical analyses were calculated using R (Version 4.1.3) and R Studio (Version 2021.09.1). The R package psych (41) was used to calculate the descriptive statistics of the mHEI-2015 and HEI-2015. To create the Bland–Altman plot, the R packages ggplot2 (42), ggExtra (43), and blandr (44) were used.

To illustrate the comparability of the calibrated mHEI-2015 with the HEI-2015 at the individual level, scores of six diets were calculated. Based on Turner-McGrievy et al. (45), data from the US news and World Report was extracted, which conducts an annual report on popular diets (46). We selected six evidence-based diets, which were included in the American Diabetes Association Consensus report (47). Diets selected for illustration included the Mediterranean, DASH, vegan, vegetarian, ketogenic, and Paleo diet. Each of these diets includes a sample menu for which the mHEI-2015 and HEI-2015 were calculated using the created metric and original FPED 2017-2018.

## Results

### The development of the database methodology

A metric database methodology was developed based on the FPED model (37). As in the FPED 2017-2018, equivalents of the fruit, vegetable, and grain components are calculated based on the processing state. For the dairy component, the metric methodology uses the exact, rather than approximate, calcium content as the basis for calculating the component. The equivalents of the protein food components (total protein foods and seafood and plant proteins) have a different calculation basis in the FPED 2017-2018 and in the metric methodology presented here. In the FPED 2017-2018, ounce equivalents are calculated based on the fat-free portion and processing state. This type of calculation requires nine different formulas depending on the subcategory as well as detailed information on the processing yield. To make the metric methodology less complex and easy to apply to other food databases, the equivalents for protein food components are calculated based on their protein content. To further increase transparency, consumption amounts for the dairy and protein food components are expressed at the nutrient level rather than in weight equivalents as in HEI-2015.

**FIGURE 2:**
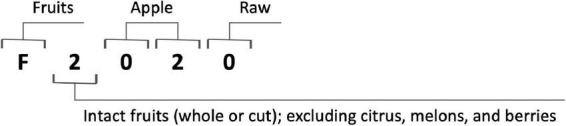
Example of the coding system using a raw apple.

For implementation of the database methodology, a five-digit coding system containing all information needed to calculate the mHEI-2015 for a weighed food record was developed. The allocation to a component of the mHEI-2015 and the processing state was clearly defined *via* the coding system. The coding system was only applicable to single food items, such as apples. For menu components such as apple pies, an additional recipe database is required to break them down into ingredients. In addition, the system can only be applied to food databases that contain information on energy, protein, fatty acids, added sugars, sodium, and calcium content. The code for a raw apple can be found as an example in Figure 2.

The **first digit** describes the type. It determines whether a food belongs to the fruit, vegetable, grain, protein, or dairy component by the first digit of the components name. If a food such as water does not belong to any component, the first digit is an “X” and no other digits follow.

The **second digit** specifies the group. Its meaning depends on the first digit. All possible combinations and their meaning are listed separately (Supplementary Table 1). Some of the differentiations in the FPED are not needed for the HEI-2015 but allow a further development of the HEI.

The **third and fourth digits** describe the individual food item. Foods that share the same first two digits are numbered consecutively. The allocation can be made, according to alphabetical order. The raw form of a food and its processing states share the same 3rd and 4th digit. For the protein foods and the dairy component only the two first digits are needed since their gram equivalents are computed by protein and calcium content exclusively.

The **fifth digit** defines the processing state of the food. Its expression can be either “raw” (0) or “processed” (1). The status “processed” does not need to be further differentiated, since the gram equivalents are calculated *via* the change in energy density. Menu components are scored proportionally to their components and do not fall under the status “processed.”

Based on the five digit-code, the gram equivalents can be calculated and the nutrient content of protein foods and dairy can be allocated. To calculate the gram equivalents the consumed amount of a food is multiplied by a factor. The factor depends on the processing state and the nutrient content. All raw foods, with the exception of protein foods, dairy products and legumes, are rated at a factor of one. In the FPED 2017-2018 methodology, cooked legumes were scored the same as raw vegetables (37) and therefore are also an exception in the metric methodology. Thus, cooked legumes receive a factor of one, while uncooked legumes have a higher factor due to their higher energy density. Below are the formulas for the factors used to convert grams to gram equivalents.

**Foods in their raw form:**


f⁢a⁢c⁢t⁢o⁢r=1

**Foods in their processed form:**


$$f\,a\,c\,t\,o\,r=\frac{e\,n\,e\,r\,g\,y\,d\,e\,n\,s\,i\,t\,y\,i\,n\,p\,r\,o\,c\,e\,s\,s\,e\,d\,f\,o\,r\,m}{e\,n\,e\,r\,g\,y\,d\,e\,n\,s\,i\,t\,y\,i\,n\,r\,a\,w\,f\,o\,r\,m}$$

### Application of the methodology to the Food Patterns Equivalents Database

The methodology was applied to both the FPID 2017-2018 for calibration and the FPID 2015-2016 for comparison. The two databases together contain 2,781 different foods, including 1,531 individual foods to which the methodology was directly applied. Recipes were reconstructed for 1,260 menu components. The amount of strawberries contained in 100 g of strawberry yogurt, for example, was calculated by dividing the cup equivalents per 100 g in the corresponding component. According to FPID 2017-2018, strawberry yogurt (Yogurt, Greek, strawberry, lowfat) contains 0.03 cup equivalents of berry fruit per 100 g. Raw strawberries count as 0.69 cup equivalents berry fruit per 100 g. This results in 4.3% berry fruit or 4.3 g of strawberries in strawberry yogurt per 100 g. As a raw fruit, strawberry has a gram equivalent of 1 in the metric methodology, so strawberry yogurt has 4.3 g berry equivalents in the metric FPID. With the metric FPID the gram equivalents for all 8899 FPED foods were computed. Discrepancies between the FNDDS ingredients database and the FPED that could not be attributed to rounding were found for 52 recipes. As example the food “lettuce, raw” would have a rounded cup equivalent of 0.72 for dark green vegetables (V1) and 0.46 for other vegetables (V6) according to the recipe in the FNDDS ingredients database. However, Table 1 shows, the cup equivalents for “lettuce, raw” in the FPED are calculated as if it consisted of 100 % “lettuce, iceberg, raw.” The effects of these discrepancies on the comparison between HEI-2015 and mHEI-2015 are to be discussed.

**TABLE 1:** Example of a discrepancy in food databases with implications for comparison.

Menu component	V1	V6		Recipe	Ingredients	V1	V6
Lettuce, raw	0.00	0.91	≠	50%	Lettuce, iceberg, raw	0.00	0.91
				50%	Lettuce, green leaf, raw	1.43	0.00

### Calibration of Healthy Eating Index-2015 scoring standards to metric scoring standards

Using the calculated component scores of the HEI-2015 of the NHANES 2017-2018 cohort, an equivalent metric consumption recommendation for the maximum score of each component of the mHEI-2015 could be calculated using the calibration. The results of the calibration were rounded to the nearest gram equivalent for the corresponding components and to one decimal place for the protein and dairy components. The metric scoring standards of the mHEI-2015 calculated by the calibration are shown in Table 2 alongside the corresponding imperial scoring standards from HEI-2015 (27). As an example of calibration, the calculation for total vegetables is detailed here. The example calculation is shown with rounded values for better clarity. In the actual calibration, the result was rounded first. The imperial standard for the maximum score for the component total vegetables is 1.1 cup equivalents per 1000 kcal. For the NHANES 2017-2018 cohort, an average consumption of 0.71 cup equivalents per 1000 kcal was calculated, which is 64.4% of the standard for the maximum score. Using the metric methodology, 103 g equivalents were calculated for the same component and cohort. Assuming that these 103 g equivalents also correspond to 64.4% of the recommendation, the 100% after rounding results in 160 g equivalents per 1000 kcal as the metric standard for the maximum score.

**TABLE 2:** mHEI-2015 and HEI-2015 (27) components, point values, and standards for scoring.

Component	Maximum score	mHEI-2015 standard for maximum score^d^	HEI-2015 standard for maximum score^d^	mHEI-2015 standard for minimum score^d^	HEI-2015 standard for minimum score^d^
**Adequacy**					
Total fruits	5	≥141 g equivalents	≥0.8 cup equivalents	No total fruits	
Whole fruits	5	≥60 g equivalents	≥0.4 cup equivalents	No whole fruits	
Total vegetables	5	≥160 g equivalents	≥1.1 cup equivalents	No total vegetables	
Greens and beans	5	≥29 g equivalents	≥0.2 cup equivalents	No greens and beans	
Whole grains	10	≥31 g equivalents	≥1.5 oz equivalents	No whole grains	
Dairy	10	≥412 mg calcium	≥1.3 cup equivalents	No dairy	
Total protein foods	5	≥15.6 g protein	≥2.5 oz equivalents	No total protein	
Seafood and plant proteins	5	≥3.3 g protein	≥0.8 oz equivalents	No seafood and plant proteins	
Fatty acids	10	(PUFAs^a^+MUFAs^b^)/SFAs^c^ ≥ 2.5		(PUFAs^a^+MUFAs^b^)/SFAs^c^ ≤ 1.2	
**Moderation**					
Refined grains	10	≤32 g equivalents	≤1.8 oz equivalents	≥76 g equivalents	≥4.3 oz equivalents
Sodium	10	≤1.1 g		≥2.0 g	
Added sugars	10	≤6.5 % of energy		≥26 % of energy	
Saturated fats	10	≤8 % of energy		≥16 % of energy	

^a^PUFAs = polyunsaturated fatty acids; ^b^MUFAs = monounsaturated fatty acids; ^c^SFAs = saturated fatty acids; ^d^per 1.000 kcal.

### Comparison of the Healthy Eating Index-2015 and metric Healthy Eating Index-2015

To demonstrate comparability for larger populations, the HEI-2015 and mHEI-2015 were calculated for the NHANES 2015-2016 cohort as described in the methods. Total scores as well as categories of indices differ only slightly from each other (Table 3). The lowest correlation between a component of the mHEI-2015 and HEI-2015 was found in the total protein foods component. In addition, a larger discrepancy was detected in the median of the greens and beans component of the indices.

**TABLE 3:** Differences and associations of changed components and total index score of HEI-2015 and mHEI-2015 of the NHANES 2015-2016 (*n* = 7,922).

Component	Maximum score	Mean ± SD	Median (IQR)	Correlation
HEI-2015 total fruits	5	2.5 ± 2.0	2.4 (4.7)	0.975
mHEI-2015 total fruits		2.5 ± 2.0	2.4 (4.7)	
HEI-2015 whole Fruits	5	2.5 ± 2.2	2.6 (5.0)	0.988
mHEI-2015 whole fruits		2.6 ± 2.2	2.7 (5.0)	
HEI-2015 total Vegetables	5	2.9 ± 1.5	2.9 (2.8)	0.975
mHEI-2015 total vegetables		3.0 ± 1.5	2.9 (2.8)	
HEI-2015 greens and beans	5	1.8 ± 2.1	0.3 (4.7)	0.920
mHEI-2015 greens and beans		1.9 ± 2.1	0.9 (4.6)	
HEI-2015 whole grains	10	2.8 ± 3.2	1.7 (4.7)	0.987
mHEI-2015 whole grains		2.9 ± 3.2	1.7 (4.7)	
HEI-2015 dairy	10	5.5 ± 3.2	5.3 (5.8)	0.989
mHEI-2015 dairy		5.5 ± 3.2	5.4 (5.8)	
HEI-2015 total protein foods	5	4.3 ± 1.2	5.0 (1.1)	0.884
mHEI-2015 total protein foods		4.3 ± 1.2	5.0 (1.2)	
HEI-2015 seafood and plant proteins	5	2.6 ± 2.2	2.6 (5.0)	0.980
mHEI-2015 seafood and plant proteins		2.5 ± 2.2	2.2 (5.0)	
HEI-2015 refined grains	10	5.6 ± 3.6	5.9 (6.7)	0.959
mHEI-2015 refined grains		5.6 ± 3.6	6.0 (6.8)	
HEI-2015	100	52.5 ± 13.5	51.5 (18.9)	0.990
mHEI-2015		52.6 ± 13.2	51.8 (18.4)	

Metric and thus non-modified components are not listed (saturated fatty acids, fatty acid ratio, sodium and added sugar).

**FIGURE 3:**
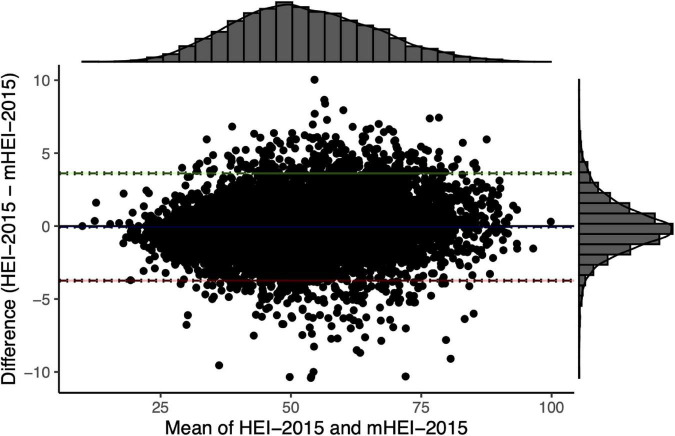
Bland–Altman plot for comparison of HEI-2015 and mHEI-2015 of NHANES 2015/2016 (*n* = 7922). The histograms display the distribution of the mean and the difference of the total scores of the two indices. The blue line represents the mean difference (–0.06 [–0.02; –0.10]). Green (3.62 [3.55; 3.69]) and red line (–3.74 [–3.81; –3.67]) are the limits of agreement with 95% confidence intervals.

The illustrated comparison of the mHEI-2015 and HEI-2015 by using the Bland-Altman plot showed good agreement with each other (Figure 3). The mean difference (bias) in total scores of the HEI-2015 and mHEI-2015 was −0.06 points (LOA 95% −3.74–3.62). Therefore, the mHEI-2015 rated the 2015-2016 NHANES marginally better than the HEI-2015 (Figure 3 and Table 3).

**FIGURE 4:**
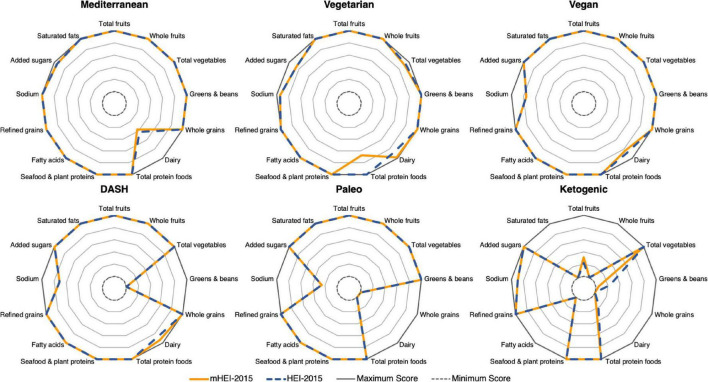
Radar graphs of HEI-2015 and mHEI-2015 for six different dietary patterns. The HEI-2015 and mHEI-2015 lines indicate the percent compliance with the recommendation of the components. Overconsumption of individual components is not shown in the figure.

Figure 4 shows the comparability of the mHEI-2015 and the HEI-2015 across different dietary patterns. Therefore, menu plans of the US news and World report of six different diets with high and low scores were evaluated. The radar graphs show the HEI-2015 and mHEI-2015 components for the six menu plans. The outer line of the radar graphs represents the maximum score of the component (100%). Depending on the component, the score is either five or ten points. The inner line represents the minimum score of 0 and thus 0%. There is no significant variation at the level of the individual components of the mHEI-2015 and HEI-2015. The largest deviation can be observed in the total protein foods component for the vegetarian daily plan, which is due to the simplified methodology of the mHEI-2015. In the mHEI-2015, the total protein foods are calculated purely based on the protein content of the foods included in the component. In the HEI-2015, nuts and seeds are weighted higher.

## Discussion

The main finding of the present study was that the newly developed mHEI-2015 showed a high degree of comparability to the HEI-2015 despite a simplified and metric methodology. The logic of cup and ounce equivalents was transferred to the metric system and gram equivalents were developed using an easily reproducible and transparent methodology. These gram equivalents are based on changes in energy density through processing and prevent over- or underestimation of food groups. Based on the HEI-2015, the determining variable for the dairy component is the calcium content and for the protein foods components the protein content is the determining variable. Therefore, food group-specific nutritional recommendations could be set for these components. Not converting to gram equivalents leads to the more transparent intake recommendation in these components. Calibration using the NHANES 2017-2018 allowed for specific metric recommendations and the closest possible approximation to the HEI-2015. Because of the conversion from volume to weight units and differences in factoring for subcategories of components, the mHEI-2015 is not an identical replica of the HEI-2015. The calculation of the cup or ounce equivalents of the FPED is partly unclear and non-transparent and includes numerous exceptions. Therefore, a comprehensive user’s guide is needed (37), which does not disclose all underlying calculations. The developed metric methodology simplifies and standardizes the calculations of gram equivalents and food group-specific components, which strongly depend on the content of individual nutrients. This is intended to make the underlying calculations as transparent as possible and to enable simple implementation without numerous exceptions. For example, while nuts and seeds are disproportionately counted in the protein food component in HEI-2015 based on their protein content (37), in the metric methodology the calculation is done for all foods based on protein content. Examination using the NHANES 2015-2016 cohort and example menu plans was able to show that the results of the two indices are transferable and comparable. Even at the individual level with different diets, similar results are obtained in each component.

In this study the comparability of the mHEI-2015 with the HEI-2015 has been shown. Construct validity, reliability, and criterion validity according to Reedy et al. are to be investigated (34). Although the components of HEI-2015 and mHEI-2015 are supported by global nutritional research and associations, results for non-U.S. populations need to be examined to confirm transferability. The developed method achieved high comparability in all components. Strong outliers in components such as greens and beans, refined grains and whole grains are due to deviations in the recipes of the FPED and FNDDS. This results in significant individual-level variation across these components and reduced component correlation. For the application to other databases based on one and not more data origins, these discrepancies are not significant. Besides these limitations, the developed methodology offers numerous advantages for countries with metric system:

1. Applicability to metric food databases,
2. An easy evaluation of metric dietary data such as weighed food records,
3. A high comparability to the HEI-2015,
4. And a metric assessment of diet quality independent of quantity following the HEI-2015.

Furthermore, the developed database methodology allows country-specific adaptations based on the mHEI-2015 with corresponding reference values. The HEI-2015 is applied worldwide for diet quality assessment (48–50). Due to unspecified information on the survey method or database, the application varies widely and is associated with different methodological problems. The use of the HEI-2015 as well as the mHEI-2015 is intended to standardize nutrition surveys due to high comparability and the same methodological origin as well as components.

The HEI is used primarily for analysis of observational studies with a large number of cases, but is also of interest for intervention studies (36). Emerging evidence supports several nutritional concepts for the treatment and prevention of obesity, diabetes mellitus type 2 or cardiovascular disease (47, 51). Individualized lifestyle interventions can address preferences by offering a variety of approaches. These nutritional concepts have overlap in critical components as found in HEI-2015 or mHEI-2015. The mHEI-2015, like other Healthy Eating Indices, can therefore be used to examine diet quality during coaching interventions for chronic disease (36), especially when implementing different nutritional approaches.

## Conclusion

A metric database methodology and metric version of the HEI-2015 was developed and reviewed for agreement with the original HEI-2015 using imperial units. This allows easy application in combination with metric food databases and metric dietary data. The mHEI-2015 allows for superior analysis of metric dietary data to better examine the relationship between chronic diseases and diet. In the future, the mHEI-2015, like the HEI-2015, should be comprehensively evaluated according to Reedy et al. (34) to show that it is associated with similar health outcomes, despite its high degree of comparability. In addition, use in intervention studies to evaluate dietary change in various nutritional approaches is planned (52).

## Data availability statement

The original contributions presented in this study are included in the article/Supplementary material, further inquiries can be directed to the corresponding author.

## Ethics statement

The studies involving human participants were reviewed and approved by the CDC NCHS Research Ethics Review Board (Protocol #2011-17 and #2018-01). Written informed consent to participate in this study was provided by the participants’ legal guardian/next of kin.

## Author contributions

JK, VH, PH, CL, OF, AG, and DK contributed to the conception and design of the study. PH organized the database and performed the calibration. JK performed the statistical analysis and wrote the first draft of the manuscript. VH, PH, CL, OF, AG, and DK read and commented critically on the manuscript. All authors contributed to the manuscript revision, read, and approved the submitted version.
