# Supplementary material for: Development of a metric Healthy Eating Index-2015 and comparison with the Healthy Eating Index-2015 for the evaluation of dietary quality
Source: Front Nutr. 2022 Aug 23;9:952223. doi: 10.3389/fnut.2022.952223 (PMC9448016; doi:10.3389/fnut.2022.952223)
Supplement: Supplementary file 1 [file Table_1.DOCX]

Supplementary Material

# Supplementary Data

Table 1: Types and groups in the coding system

| F1 | Intact fruits (whole or cut) of citrus, melons, and berries |
| --- | --- |
| F2 | Intact fruits (whole or cut); excluding citrus, melons, and berries |
| F3 | Fruit juices, citrus and non citrus |
|  |  |
| V1 | Dark green vegetables |
| V2 | Tomatoes and tomato products |
| V3 | Other red and orange vegetables, excluding tomatoes and tomato products |
| V4 | White potatoes |
| V5 | Other starchy vegetables, excluding white potatoes |
| V6 | Other vegetables not in the vegetable components listed above |
| V7 | Legumes computed as vegetables |
|  |  |
| G1 | Whole grains |
| G2 | Refined or non-whole grains |
|  |  |
| P1 | Beef, veal, pork, lamb, game meat; excludes organ meats and cured meat |
| P2 | Cured/luncheon meat made from beef, pork, or poultry |
| P3 | Organ meat from beef, veal, pork, lamb, game, and poultry |
| P4 | Chicken, turkey, Cornish hens, and game birds; excludes organ meats and cured meat |
| P5 | Seafood (finfish, shellfish and other seafood) high in n-3 fatty acids |
| P6 | Seafood (finfish, shellfish and other seafood) low in n-3 fatty acids |
| P7 | Eggs (chicken, duck, goose, quail) and egg substitutes |
| P8 | Soy products, excluding calcium fortified soy milk and immature soybeans |
| P9 | Peanuts, tree nuts, and seeds, excludes coconut |
| P0 | Legumes computed as protein foods |
|  |  |
| D1 | Fluid milk and calcium fortified soy milk |
| D2 | Yogurt |
| D3 | Cheese |
